# Using interactive multimedia e-Books for learning blood cell morphology in pediatric hematology

**DOI:** 10.1186/s12909-016-0816-9

**Published:** 2016-11-14

**Authors:** Chih-Cheng Hsiao, Mao-Meng Tiao, Chih-Cheng Chen

**Affiliations:** 1 Division of Hematology/Oncology, Department of Pediatrics, Kaohsiung Chang Gung Memorial Hospital and Chang Gung University College of Medicine, 123 Tai-Pei Road, Niao Sung, Kaohsiung, Postal code: 833 Taiwan; 2 Division of Gastroenterology, Department of Pediatrics, Kaohsiung Chang Gung Memorial Hospital and Chang Gung University College of Medicine, Kaohsiung, Taiwan; 3 Division of Neonatology, Department of Pediatrics, Kaohsiung Chang Gung Memorial Hospital and Chang Gung University College of Medicine, Kaohsiung, Taiwan; 4 Chang Gung Memorial Hospital Research Centre for Medical Education, Taipei, Taiwan

## Abstract

**Background:**

This prospective study compares the use of interactive multimedia eBooks (IME) with traditional PowerPoint (TPP) for teaching cell morphology of blood and bone marrow.

**Methods:**

Fifty-one interns from three Taiwan medical schools training by a single teacher in the pediatric hematology department of Kaohsiung Chang Gung Memorial Hospital, Taiwan, participated in this study. 25 interns were allocated for training with a traditional PowerPoint atlas and 26 interns for training with an interactive multimedia eBook atlas. Learning outcomes were examined by pre-test and post-test using the CellQuiz of CellAtlas App. Attitudes and perceptions were collected by survey questions regarding interest, motivation and effectiveness.

**Results:**

There was no difference in the pre-test scores between TPP and IME groups (mean score 27.0 versus 27.9, *p* = 0.807). However, the interns in the interactive multimedia eBook group achieved significantly better scores in the post-test than the ones in the PowerPoint group (mean score 103.2 versus 70.6; *p* < 0.001). Overall results of interest, motivation and effectiveness were strongly positive in the multimedia eBook group.

**Conclusions:**

Our data supports that interactive multimedia eBooks are more effective than PowerPoint to facilitate learning of cell morphology of blood and bone marrow.

**Electronic supplementary material:**

The online version of this article (doi:10.1186/s12909-016-0816-9) contains supplementary material, which is available to authorized users.

## Introduction

E-learning offers many advantages over conventional teaching methods. A recent review of e-learning medical education reported that learners are more likely to be engaged when the technology is easy to use [1–4]. There is the potential to utilize multimedia applications with interactivity. It allows users to learn at their own pace as well as at their own convenience. The basis for this interactive e-learning research is to increase student learning interest and efficiency and allow students to more fully comprehend the basics necessary for clinical practice. We hope that interactive e-learning provides a higher motivation for learners by presenting content in an interactive, game-based and competitive environment. E-learning is associated with greater learning efficiency, problem solving abilities and satisfaction [5, 6].

In recent years, there has been increasing emphasis of active learning and self-directed learning in medical education curriculum [7, 8]. Schneider et al. created computer-based cases for important diagnoses in urology using the authoring system CASUS® [9]. They found that members of the CASUS® group using the online cases for preparation scored significantly higher with an average of 20% better test results than students using textbooks for preparation. They confirmed that e-learning resulted in an interesting and engaging learning model with improved learning outcomes. In the study of Boeker M et al., comparing the effectiveness on the learning outcome of a Game-based e-learning instruction with a conventional script-based instruction in the teaching of microscopy urinalysis for medical students [10]. In this study, the students in the Game-based e-learning group achieved significantly better results in the cognitive knowledge test than the students in the script group. Attitudes also towards the recent learning experience were significantly more positive with Game-based e-learning group. Currently, E-learning has a major impact on the effectiveness of learning and provides students to active learning and self-directed learning. However, development of good e-learning materials including multimedia, simulation or game-based resource requires a multidisciplinary team and can be resource intensive [10–13]. To achieve a highly interactive and active learning experience for the student, not only considerable programming is required by the instructional teams, but also ensuring that the time and money invested is justified by institutions on many impacts. The concepts were perceived by previous reports [5, 6]. Therefore, the development and promotion toward interactive e-learning has shown limitation in the medical education.

Using traditional teaching methods to teach interns hematology, one issue with students was that the materials used did not effectively train students to differentiate blood cells, when they started to study in the pediatric hematology department, because the knowledge delivered was limited. Also, many students find the PPT materials a little dry, and we hoped to create more engaging materials for students. The creation of an interactive eBooks has the potential to transform learning away from basic acquisition of facts (i.e. printed materials, didactic lectures, PPT) to actively acquiring and applying knowledge and skills as it can include multimedia and interactive learning.

In this study, SimMAGIC eBooK editing software (http://www.simmagic.com.tw/Products/eBook/intro.aspx. com.tw/Products/eBook/functions.aspx) was employed to create an interactive multimedia eBook of blood cell morphology. This eBook software uses a simple and clear editing layout and includes customized editing tools and various content editing functions enabling editors to make creative content for eBooks and publications quickly. In addition, it emphasizes interactive quiz creation and easy design by integrating and importing various multimedia, such as PPT, PDF or video to make a simulative and operational eBook. Students interact with the operational eBook very well. In addition, eBook includes many different operation simulations, allowing students to not only enjoy reading but also interact with the book and take simulated tests. Customize right/wrong answer pop-up message windows, simulative interactive functions such as input, click and drag, screen clicking and selection, also include links to related documents providing additional support. Students could get feedback immediately and learn effectively.

This study compares the use of interactive multimedia eBooks with traditional PowerPoint for teaching cell morphology of blood and bone marrow. We wanted to know whether eBooks lead to an improved outcome and how students react to interactive eBooks. With regards to learning efficiency, student engagement and appeal, our study was to determine if eBooks are superior to a conventional instructional method for medical students learning blood cell morphology. Additional data on students’ feedback towards both TPP and IME learning was collected from their respective groups, including attitudes towards the course material, enjoyment, intuitiveness, learning methods and confidence.

## Methods

**Fig. 1:**
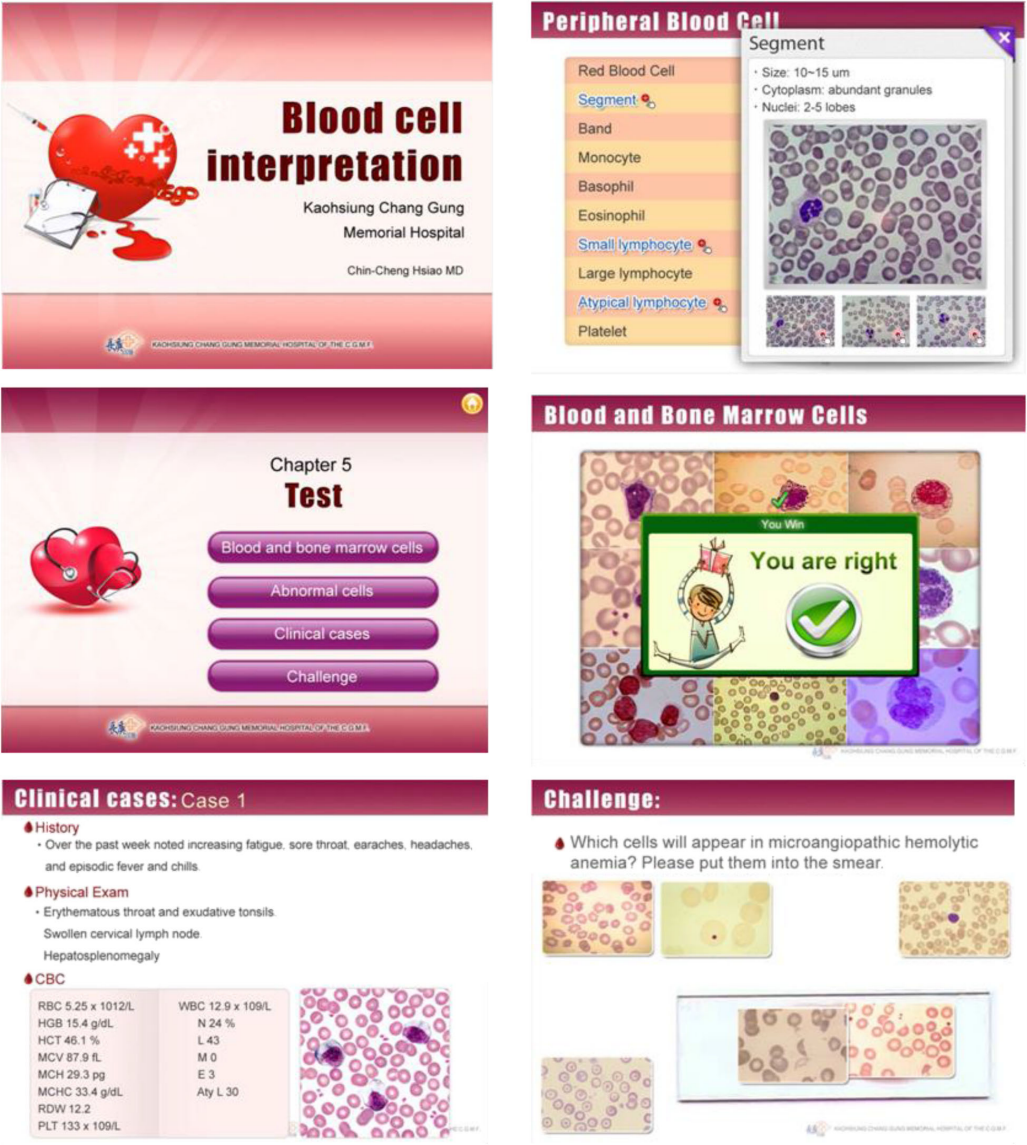
Using SimMAGIC eBook software to create interactive multimedia eBooks of cell morphology of blood and bone marrow. The eBooks were specifically created for this study

**Fig. 2:**
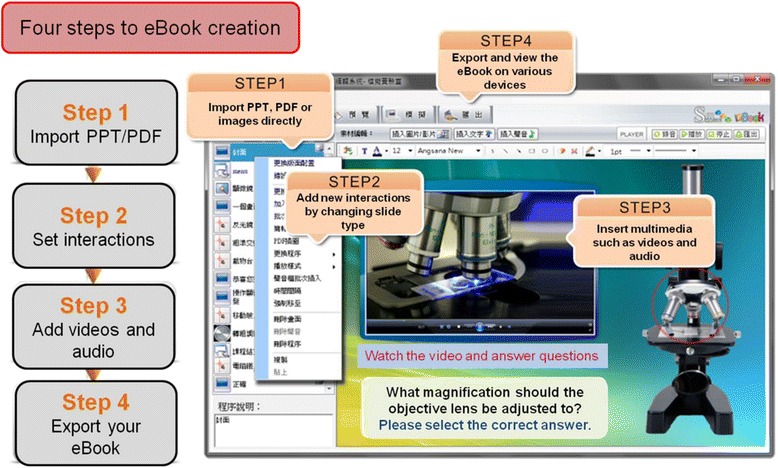
Create an eBook in Four Easy Steps: step 1. Import PPT/PDF; step 2. Setting interactions; step 3. Add videos and audio; step 4. Export eBook

In this study, we used SimMAGIC eBook software (Hamastar technology company, Taiwan. http://www.simmagic.com.tw/Products/eBook/intro.aspx) to easily create interactive multimedia eBooks for learning cell morphology of blood and bone marrow (Fig. 1). SimMAGIC eBook editing software includes many different operations and simulations, allowing editors including teachers and students to use various interactive functions such as: text input, page jumping, mouse dragging, picture clicking, error message link choices and many others to create eBooks (Fig. 2). The saved working file can always be edited or updated, and anyone can use the eBook creator. Moreover, it also integrates multimedia objects such as PPT, PDF, images, video, audio and flash for editors to insert into a specialized interactive multimedia eBook in each slide to make the simulative and operational eBook contents. Readers not only enjoy reading but also interacting with the eBook and taking simulated tests, increased reading and learning participation. Furthermore, we have also established Cloud Bookshelf Management Platform that we can monitor complete records, statistical analysis of user reading and testing history, and supports recording of offline reading history allowing the administrator a grasp of the user’s reading history through matching and cross referencing reports.

This randomized controlled trial was conducted from Jan, 2013 to Dec, 2013 in the pediatric hematology department at Kaohsiung Chang Gung Memorial Hospital, Taiwan. The curriculum was composed of a 2-week course for learning pediatric hematology. Fifty-one interns participating in the study during their rotation at the Department of Pediatric Hematology were eligible for inclusion in the study. Interns that rotated to the pediatrics hematology department who were interns were selected for this study. At the beginning of each week of training, the students were told that they would participate in an educational study and that their participation would have no effect on their final grades and scores.

All interns were allocated a traditional PowerPoint atlas or an interactive multimedia eBook atlas, as their study material. Purely self-directed learning was requested. Students in the traditional PowerPoint group (TPP) learned the topic of blood cell morphology in a conventional PPT approach, while the students in the interactive multimedia eBook group (IME) learned the topic with an eBook. For the TPP approach, the students were provided with power point slides containing all the material to acquire knowledge on the learning objectives. The TPP includes an introductory part on the blood cell formation, normal cell morphology of blood and bone marrow. In the following section, the TPP scripts abnormal cells. Finally, it explains diseases in which the blood cells represent. For the IME group, the learning content was almost identical to TPP except interactive tests, the contents including five chapters. Chapter 1: Introduction to Blood Cell Formation, Chapter 2: Peripheral Blood Cells, Chapter 3: Bone Marrow Cells, Chapter 4: Recognizing Abnormal Cells, Chapter 5: Interactive Game-based Tests. IME include four types of interactive game-based practice: correctly identifying cell types, identifying abnormal cell types, clinical case examination and challenges. The student interactive learning included picture matching, multiple choice, clinical diagnosis simulations, picture labelling and drag and drop e-learning modules. Students can see their rank and progress.

The eBook includes functionality for automated marking, allowing the system to indicate to the user if their chosen annotated answer was correct or incorrect. The reasoning behind the answer was also provided to the user, regardless of the answer outcome, therefore students could further understand the content, especially when incorrect answers were given. The student answers and data was sent to a server to centralize the data for the teacher, and by analyzing the class progress and results, the teacher could adjust his classes accordingly, responding to problem areas revealed by the captured in the server data.

**Fig. 3:**
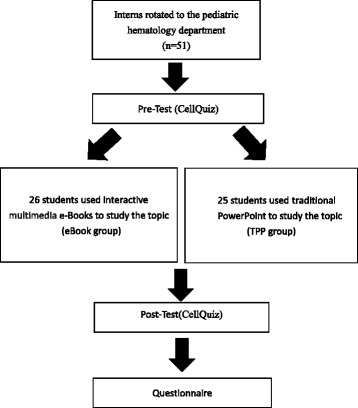
Flow chart of the study. A total of 51 interns were randomized to eBook group and TPP group

Learning outcomes were examined by pre-test and post-test using the CellAtlas App’s CellQuiz, an app to test users regarding cell morphology identification. CellAtlas is a product from the medical technology company CellaVision (http://www.cellavision.com/en/cellavision-cellatlas). The online cell image database was launched in 2000. The lectures provide a basic knowledge of the wide field of cell morphology. Each section contains a brief summary and additional links provide further explanations and additional images. CellaVision provides an App that is made up of two parts. The first is CellAtlas, which contains mini lectures written by hematology experts complemented by an extensive cell image database and content including hematopoiesis, peripheral leukocytes and erythrocytes, and other findings in peripheral blood. The second part of the App is CellQuiz, a quiz-based game that tests the user’s knowledge in identifying cell types, which matched our learning goals for this study. It is noted that CellAtlas was not used in our study, but CellQuiz was used in our study for our cell identification pre-test and post-test scoring. CellQuiz allows users one minute to identify cell types. A question has nine pictures, and a user is given three tries to make a correct identification. A question gives 20 points and each error deducts 5 points. If the user incorrectly chooses three times for a given question, the user is deducted 15 points, and the quiz moves onto to the next question. Figure 3 shows a flow chart of the study.

A feedback questionnaire comprised of five questions was used to record students’ perceptions on the utilization of different learning resource materials. Data was gathered using a five-point Likert scale (Table 1).

**Table 1:** Students’ Feedback Questionnaire

No.	Statement	Students’ rating	
		TPP	IME
1	Would you like to have more learning material like this in your training?	3.16 ± 1.11	4.50 ± 0.65
2	Did you enjoy studying with the learning material?	3.36 ± 0.64	4.69 ± 0.68
3	Did you think the learning method of delivery helped my core skills?	3.08 ± 0.64	4.73 ± 0.60
4	Was this kind of learning intuitive and user friendly?	2.80 ± 0.60	4.58 ± 0.76
5	Do you feel confident in the domain of blood cell morphology now?	3.08 ± 0.95	4.58 ± 0.64

Values of students’ rating (Five-point Likert scale is used, where 1—Strongly Disagree, 2—Disagree, 3—Neutral, 4—Agree, 5—Strongly Agree) are represented as mean ± SD

Statistical methods employed were descriptive statistics and student’s *t*-test in comparison between TPP and IME groups. A *p*-value < 0.05 is considered statistically significant. The statistical analyses were performed using the Statistical Package for Social Science (SPSS, version 18) software package.

## Results

**Fig. 4:**
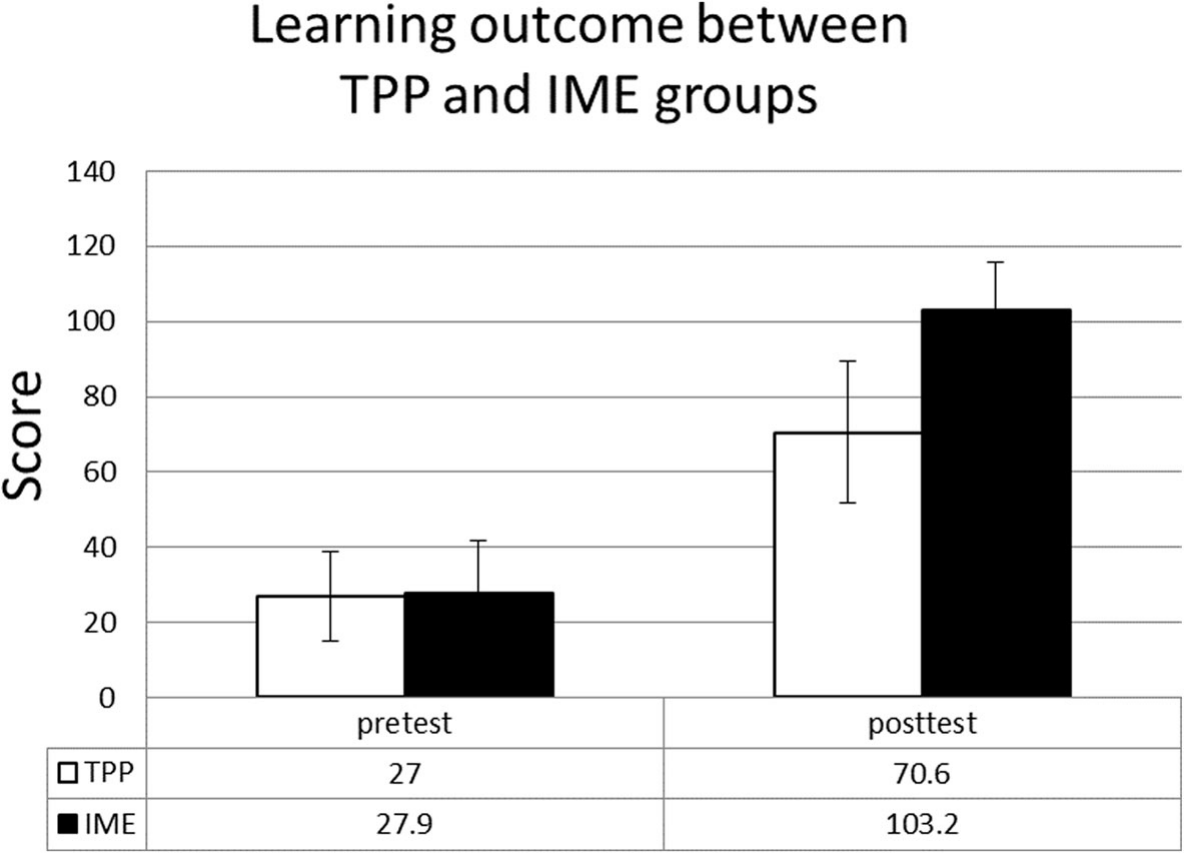
The interns in the interactive multimedia eBook (IME) group achieved significantly better scores in the posttest than the ones in the PowerPoint (TPP) group with a mean score of 103.2 ± 13.6 and 70.6 ± 13.7, respectively (*p* < 0.001). Descriptive statistics and student’s *t*-test were used in comparison between TPP and IME groups. A *p*-value < 0.05 is considered statistically significant

Fifty-one students consented to take part in this study (*n* = 51). 25 interns were allocated a traditional PowerPoint atlas for training, and 26 interns were allocated an interactive multimedia eBook atlas. As mentioned, the pre-test and post-test use CellQuiz, which allows the students only one minute to identify cell types. A user is given three tries to make a correct identification. A question gives 20 points and each error deducts 5 points. If the user incorrectly chooses three times for a given question, the user is deducted 15 points. The mean score and standard deviation for the group using the traditional PowerPoint group was 27(11.9) and mean score for the eBook group was 27.9(18.8). There was no difference in the pretest score between TPP and IME groups (*p* = 0.807) (Fig. 4). However, the interns in the interactive multimedia eBook group achieved significantly better scores in the posttest than the ones in the PowerPoint group with a mean score and standard deviation of 103.2(13.6) and 70.6(13.7), respectively (*p* < 0.001) which was an increase of 269.9% for the eBook group, and 161.5% for the PowerPoint group. Overall, the eBook group achieved a mean score which was higher than the PowerPoint group by 46.2%.

**Fig. 5:**
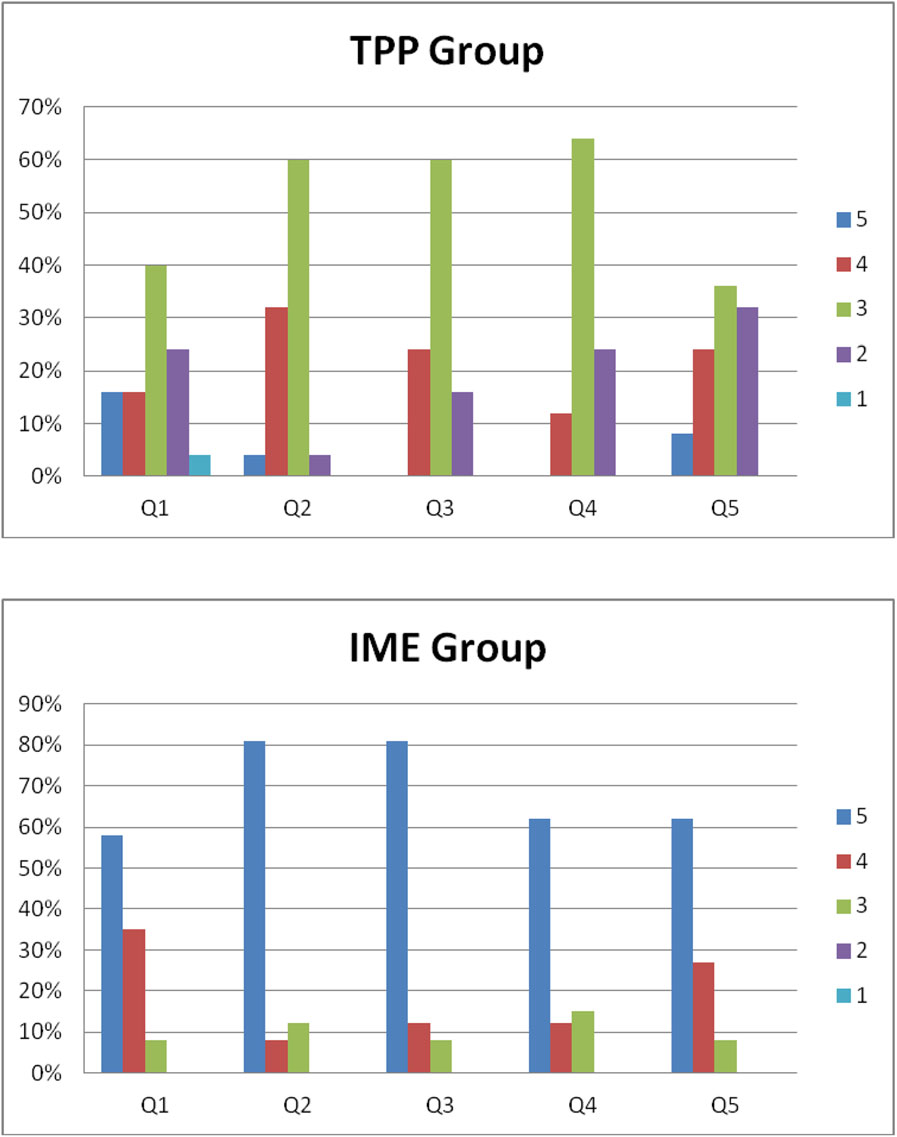
Five-point Likert scale is used, where 1 Strongly Disagree, 2 Disagree, 3 Neutral, 4 Agree, 5 Strongly Agree. A five-point Likert scale questionnaire was used to gather data regarding multiple modes of representation and students’ attitudes toward interactive multimedia eBook and traditional PowerPoint

At the end of the study, a five-point Likert scale questionnaire was used to gather data regarding multiple modes of representation and students’ attitudes toward interactive multimedia eBook and traditional PowerPoint. Mean scores for all Likert scale questions are listed in Table 1. Within our study, students responded to the question, “Would you like to have more learning material like this in your training?” only 32% of participants in the TPP group answered”agree” versus 92.3% in the IME group. For the question, “Did you enjoy studying the provided learning material?” in the TPP group, only 36% chose “agree”, compared to 88.5% in the IME group. When asked “Did you think the learning method of delivery helped my core skills?” only 24% answered “agree” in the TPP group versus 92.3% in the IME group. Furthermore, when asked “Was this kind of learning intuitive and user friendly?” 12% chose “agree” in the TPP group versus 84.6% in the IME group. Finally, when asked the question,”Do you feel confident in the domain of blood cell morphology now?” 32% in the TPP group answered “agree”, compared to 92.3% in the IME group (Fig. 5).

## Discussion

This study compares two learning methods used to teach blood and bone marrow cell morphology, one being a more traditional way of learning using PowerPoint and the other method using eBooks. There was no instruction given to students. Both groups were asked to review the course material given. In the TPP group, students viewed the power point slide materials, while the eBook group view the eBook materials provided. The two groups had similar pre-test scores. However, it was found that a significantly better outcome was achieved by participants in the eBook group in the post-test scores. The learning content in IME group was almost identical to TPP group, except IME included interactive game-based tests. This could be due to the fact that the eBook provided interactive tests for participants to use, compared to none for the PowerPoint group. With our randomized controlled trial, this might explain the different test outcomes between the two groups and the results could also mean that learning with interactive multimedia eBooks is not only more effective, but also more attractive to students than learning through traditional methods. Furthermore, it is important to note that students were interested in learning blood cell morphology even though it meant investing more time in practicing tests, and this also demonstrates the high acceptance of eBook in this study.

Our study not only shows that students who were provided with eBook resources had a significantly higher score when compared with the students who learned the same material through PPT slides. Additionally, the participants in the eBook group indicated higher Likert scores when answering if they enjoyed the learning materials and methods provided, if they would like to learn more using this style of teaching, and if they were more confident in regards to their knowledge of the topic. The IME group was not given CellQuiz to practice raising their scores, and was only used in the pre-test and post-test portion of this research. Our research was primarily to compare our existing training course, which only uses PowerPoint slides, with a new teaching platform, eBooks, to compare student learning and engagement, which more fully uses the functionality of computer systems and newer cloud technologies. Through our study, we found that students performed better in the IME group, and questionnaire scores indicated that students preferred the eBook platform. With our study, we provide clear evidence that eBooks have a beneficial effect on the students’ knowledge and their attitudes towards their learning experience.

A bottleneck might occur in TPP group because their learning materials only have cell pictures and texts. Through the questionnaire used in this study, it was found that TPP students were not as engaged in the learning process. In the IME group, we not only provided many interactive game-based tests, but also immediate feedback in the form of automated marking and progress reports. Teachers would also know the progress and performance of the class and students, allowing for a feedback system relating to the overall course progression. This included the complete records of each student, statistical analysis of user usage and testing history from Cloud Bookshelf Management Platform.

The Likert survey results show that multiple modes of representation and multimedia elements are strongly supported by students when comparing to traditional methods of delivery. Students in our study favorably responded to the addition of interactive elements in the eBook and believed that these elements aided their learning for recognition of blood cell morphology. Interaction is a useful tool for training medical students in core competencies. Stirling A and Birt J (2013) used interactive eBooks to represent anatomy and identification through additional 3D modeling software and imaging. They compared the use of an enriched multimedia eBook with traditional methods for teaching gross anatomy of the heart and great vessels. They found initial interaction with multimedia content followed by active experimentation in the anatomy laboratory led to improved performance in the final test [14]. Furthermore, additional literature has shown that using various e-learning tools such as video, multimedia, internet interactive modules could improve core skills and deliver concepts in clinical medicine [5, 15]. The goal of this study was to allow interns to increase their cell identification skills while rotating to the pediatrics hematology department. The core skill of cell identification is a basic skill required if doctors hope to work in this field of medicine.

Developing good simulations, game-based learning and other e-learning materials such as virtual reality can be resource intensive, and it is therefore important to ensure that the time and money invested is justified by the outcome. In this eBook program, we sought to employ technology in the form of interactive and multimedia sessions to promote self-directed learning among students. To enhance the interactivity of the presentation, we used SimMAGIC e-Book editing software, which provides a simple and clear editing layout, customized editing tools and various content editing functions enable editors to make creative content for the e-Books. It is noted that the cost of this software was reasonable as compared to other choices on the market. Furthermore, an e-book workshop for teachers was held in our hospital. They needed to take 9 h of lectures and practice. All teachers unanimously considered this software suitable for editing e-books in clinical education. It can be tailored to an educational institution’s requirements and produced quickly by hospital staff at a minimal cost.

We believe that in some cases, eBooks could not substitute classical teaching. However, eBook could be used in conjunction with traditional teaching methods to provide a more comprehensive learning environment. This would include standard classes with a teacher and eBooks, to allow for classroom learning through an expert and independent learning outside of class. eBooks can provide additional independent directed interactive learning outside the classroom. There is a great deal of literature emphasizing the role of technology and how it can be used as a complementary resource [9, 11, 16–19]. The authors suggest that eBooks have a place in reducing teaching load and expense when used as an adjunct to traditional methods. It provides an effective and user friendly learning tool in medical curriculum. Traditional learning could combine eBooks as the interactive learning and test-based features allow students more practice opportunities inside and outside of the classroom. The automated marking features also reduce load of the teachers. Furthermore, it permits students the ability to effectively resolve their learning deficiencies in their own time.

### Limitations

One limitation of this study was the relatively low-participant number (51 students) enrolled in the study. In addition, one could suppose that students in the eBook group spend more time in the interactive tests on the topic than the students in the PowerPoint group, and so the increased performance could be due to longer learning time invested. Study times were not collected in our study, and therefore, we were unable to gauge the amount of time students in each group spent learning the course material.

In addition, we did not control other certain factors which might have influenced the learning outcome. This could have been students learning morphology of blood and bone marrow cells outside of the practical-training time on their own. However, on the one hand, this factor is randomly distributed over both groups and, on the other hand, it might be understood as a motivational effect of the specific training, which could not be further analyzed by our study. Specifically, CellQuiz is a commercial App and students could download it easily, the score of a post-test could be affected by repeated practice, although there was no limitation on the depth and quantity of questions used in each phase of testing. However, this factor is randomly distributed over both groups and it may be understood as a motivational effect of the specific training. Regardless, this factor was not further analyzed in our study.

## Conclusions

This study compares a more traditional method of learning using PowerPoint and a second method of using interactive eBooks. It was found that a significantly better learning outcome was achieved by participants in the eBook group. One major difference between the two learning methods was that eBook provided interactive learning and tests for participants to use, whereas the PowerPoint learning material did not contain any. The different test outcomes and results between the two groups could mean that learning through interactive multimedia eBooks is not only more effective, but also more attractive to students, compared to learning through traditional methods. These higher scores also point to a high level acceptance of eBook and e-learning materials for medical students. With such strong evidence in this study that interactive learning through eBooks greatly increased learning effectiveness and scores of participants, other curriculums may also benefit with the inclusion of appropriate eBook and e-learning materials.

## Additional file

Additional file 1: The scores of pretest and posttest and five-point Likert scale questionnaire for both groups. This is raw data for analysis in this study. (PDF 107 kb)
