# Supplementary figures and images for: Using interactive multimedia e-Books for learning blood cell morphology in pediatric hematology
Source: BMC Med Educ. 2016 Nov 14;16:290. doi: 10.1186/s12909-016-0816-9 (PMC5109786; doi:10.1186/s12909-016-0816-9)

| Group A pretest | Group A posttest | 1    | 2    | 3    | 4    | 5    | Group B pretest | Group B posttest | 1   | 2        | 3        |
|-----------------|------------------|------|------|------|------|------|-----------------|------------------|-----|----------|----------|
| 10              | 80               | 3    | 4    | 4    | 3    | 3    | 30              | 100              | 5   | 5        | 5        |
| 20              | 75               | 3    | 3    | 3    | 3    | 3    | 25              | 125              | 5   | 5        | 5        |
| 25              | 60               | 3    | 3    | 3    | 2    | 3    | 20              | 115              | 5   | 5        | 5        |
| 40              | 95               | 5    | 4    | 4    | 2    | 4    | 10              | 95               | 4   | 5        | 5        |
| 30              | 60               | 3    | 3    | 3    | 2    | 2    | 35              | 90               | 4   | 5        | 5        |
| 20              | 40               | 2    | 4    | 2    | 3    | 2    | 10              | 130              | 5   | 5        | 5        |
| 35              | 75               | 3    | 3    | 3    | 3    | 3    | 50              | 100              | 5   | 5        | 5        |
| 10              | 80               | 3    | 3    | 2    | 3    | 3    | 30              | 95               | 5   | 5        | 5        |
| 15              | 40               | 2    | 4    | 3    | 3    | 2    | 20              | 85               | 4   | 3        | 3        |
| 40              | 80               | 3    | 3    | 3    | 4    | 4    | 15              | 95               | 4   | 5        | 5        |
| 30              | 85               | 3    | 5    | 3    | 3    | 4    | 40              | 110              | 5   | 4        | 4        |
| 45              | 90               | 5    | 3    | 4    | 3    | 4    | 45              | 120              | 5   | 5        | 5        |
| 20              | 40               | 2    | 2    | 2    | 3    | 2    | 60              | 120              | 5   | 4        | 5        |
| 20              | 50               | 3    | 3    | 3    | 2    | 2    | 30              | 110              | 5   | 5        | 5        |
| 25              | 70               | 4    | 3    | 4    | 3    | 3    | 15              | 95               | 4   | 5        | 5        |
| 30              | 80               | 4    | 4    | 3    | 2    | 3    | 10              | 90               | 4   | 5        | 5        |
| 30              | 40               | 1    | 4    | 2    | 3    | 2    | 40              | 100              | 5   | 5        | 5        |
| 25              | 55               | 2    | 3    | 3    | 3    | 2    | 30              | 105              | 5   | 5        | 5        |
| 45              | 80               | 4    | 3    | 3    | 2    | 4    | 35              | 90               | 4   | 3        | 3        |
| 50              | 100              | 5    | 3    | 4    | 3    | 5    | 25              | 85               | 3   | 3        | 4        |
| 20              | 65               | 2    | 4    | 3    | 4    | 3    | 20              | 105              | 4   | 5        | 5        |
| 15              | 70               | 3    | 3    | 4    | 3    | 3    | 40              | 90               | 3   | 5        | 4        |
| 5               | 65               | 2    | 4    | 3    | 3    | 2    | 15              | 105              | 5   | 5        | 5        |
| 30              | 90               | 4    | 3    | 3    | 4    | 4    | 5               | 90               | 4   | 5        | 5        |
| 40              | 100              | 5    | 3    | 3    | 3    | 5    | 30              | 110              | 5   | 5        | 5        |
| 27              | 70.6             | 3.16 | 3.36 | 3.08 | 2.88 | 3.08 | 40              | 130              | 5   | 5        | 5        |
| 11.90238071     | 18.78163997      |      |      |      |      |      | 27.88461538     | 103.2692308      | 4.5 | 4.692308 | 4.730769 |
|                 |                  |      |      |      |      |      | 13.72392633     | 13.56040617      |     |          |          |

|          |          |     |         |          |  |  |      |      |  |  |  |  |
|----------|----------|-----|---------|----------|--|--|------|------|--|--|--|--|
| 4        | 5        |     | pretest | posttest |  |  |      |      |  |  |  |  |
| 5        | 4        | TPP | 27      | 70.6     |  |  | 11.9 | 18.8 |  |  |  |  |
| 5        | 5        | IME | 27.9    | 103.2    |  |  | 13.8 | 12.7 |  |  |  |  |
| 5        | 5        |     |         |          |  |  |      |      |  |  |  |  |
| 5        | 5        |     |         |          |  |  |      |      |  |  |  |  |
| 5        | 5        |     |         |          |  |  |      |      |  |  |  |  |
| 5        | 5        |     |         |          |  |  |      |      |  |  |  |  |
| 4        | 5        |     |         |          |  |  |      |      |  |  |  |  |
| 5        | 4        |     |         |          |  |  |      |      |  |  |  |  |
| 3        | 3        |     |         |          |  |  |      |      |  |  |  |  |
| 5        | 5        |     |         |          |  |  |      |      |  |  |  |  |
| 5        | 5        |     |         |          |  |  |      |      |  |  |  |  |
| 5        | 5        |     |         |          |  |  |      |      |  |  |  |  |
| 5        | 5        |     |         |          |  |  |      |      |  |  |  |  |
| 5        | 5        |     |         |          |  |  |      |      |  |  |  |  |
| 5        | 5        |     |         |          |  |  |      |      |  |  |  |  |
| 5        | 5        |     |         |          |  |  |      |      |  |  |  |  |
| 5        | 5        |     |         |          |  |  |      |      |  |  |  |  |
| 4        | 5        |     |         |          |  |  |      |      |  |  |  |  |
| 5        | 4        |     |         |          |  |  |      |      |  |  |  |  |
| 5        | 4        |     |         |          |  |  |      |      |  |  |  |  |
| 3        | 3        |     |         |          |  |  |      |      |  |  |  |  |
| 5        | 5        |     |         |          |  |  |      |      |  |  |  |  |
| 3        | 4        |     |         |          |  |  |      |      |  |  |  |  |
| 5        | 4        |     |         |          |  |  |      |      |  |  |  |  |
| 3        | 4        |     |         |          |  |  |      |      |  |  |  |  |
| 5        | 5        |     |         |          |  |  |      |      |  |  |  |  |
| 4        | 5        |     |         |          |  |  |      |      |  |  |  |  |
| 4.576923 | 4.576923 |     |         |          |  |  |      |      |  |  |  |  |
|          |          |     |         |          |  |  |      |      |  |  |  |  |

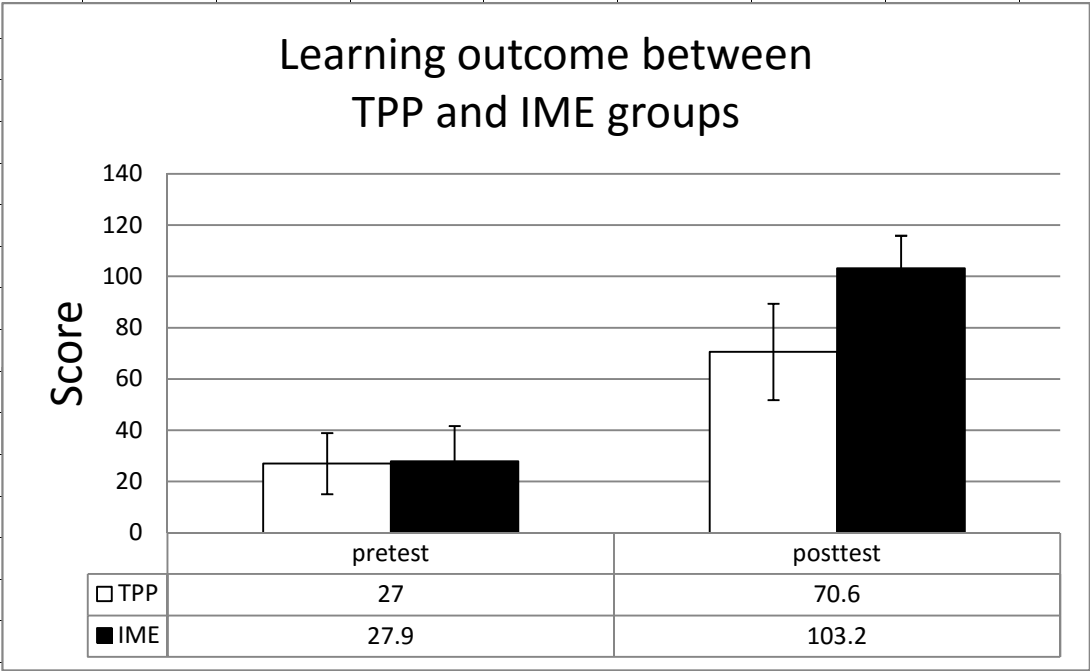

Supplement: Additional file 1: — The scores of pretest and posttest and five-point Likert scale questionnaire for both groups. This is raw data for analysis in this study. (PDF 107 kb) [file 12909_2016_816_MOESM1_ESM.pdf]
